# The Integrative Regulatory Network of circRNA, microRNA, and mRNA in Atrial Fibrillation

**DOI:** 10.3389/fgene.2019.00526

**Published:** 2019-06-13

**Authors:** Shengyang Jiang, Changfa Guo, Wei Zhang, Wenliang Che, Jie Zhang, Shaowei Zhuang, Yiting Wang, Yangyang Zhang, Ban Liu

**Affiliations:** ^1^Department of Cardiology, Seventh People’s Hospital of Shanghai University of Traditional Chinese Medicine, Shanghai, China; ^2^Department of Cardiac Surgery, Zhongshan Hospital, Fudan University, Shanghai, China; ^3^Department of Cardiology, Shanghai Tenth People’s Hospital, Tongji University School of Medicine, Shanghai, China; ^4^Department of Cardiology, Shanghai Tenth People’s Hospital Chongming Branch, Tongji University School of Medicine, Shanghai, China; ^5^Key Laboratory of Arrhythmias, Ministry of Education, Shanghai East Hospital, Tongji University School of Medicine, Shanghai, China; ^6^Basic Medical College, Jinzhou Medical University, Jinzhou, China; ^7^Department of Cardiovascular Surgery, Shanghai East Hospital, Tongji University School of Medicine, Shanghai, China

**Keywords:** atrial fibrillation, non-coding RNA, circular RNA, microRNA, mRNA

## Abstract

Atrial fibrillation (AF) is the most common irregular heart rhythm which influence approximately 1–2% of the general population. As a potential factor for ischemic stroke, AF could also cause heart failure. The mechanisms behind AF pathogenesis is complex and remains elusive. As a new category of non-coding RNAs (ncRNAs), circular RNAs (circRNAs) have been known as the key of developmental processes, regulation of cell function, pathogenesis of heart diseases and pathological responses which could provide novel sight into the pathogenesis of AF. circRNAs function as modulators of microRNAs in cardiac disease. To investigate the regulatory mechanism of circRNA in AF, especially the complex interactions among circRNA, microRNA and mRNA, we collected the heart tissues from three AF patients and three healthy controls and profiled their circRNA expressions with circRNA Microarray. The differentially expressed circRNAs were identified and the biological functions of their interaction microRNAs and mRNAs were analyzed. Our results provided novel insights of the circRNA roles in AF and proposed highly possible interaction mechanisms among circRNAs, microRNAs, and mRNAs.

## Introduction

Atrial fibrillation (AF) is the most common irregular heart rhythm which influence approximately 1–2% of the general population (Graham et al., 2015; Wang, 2018). Several important factors may increase the risk of developing AF, including age, sex, obesity, excessive alcohol consumption, hypertension, abnormal heart valves and lung diseases (Dagres and Anastasiou-Nana, 2010; Soliman et al., 2014). As a potential factor for ischemic stroke, AF could also cause hospitalization for heart failure, and death which is associated with high mortality, morbidity, and socioeconomic burden (Voukalis et al., 2016). However, current treatment of AF still lacks enough utility and efficacy which may have possibly adverse effects (Vallabhajosyula et al., 2016; Wan et al., 2016). The mechanisms behind AF pathogenesis are complex and remains elusive. Further study of the potential mechanisms of AF could provide novel treatment which could alternate current therapy effectively (Ogawa et al., 2017).

As we all know, Non-coding RNAs (ncRNAs) play important roles in regulating gene expression. The main groups of ncRNAs include long non-coding RNAs (lncRNAs), micro-RNAs (miRNAs), and circular RNAs (circRNAs) (McMullen and Ooi, 2017). As a new category of ncRNAs, circRNAs have been known as the key of developmental processes, regulation of cell function, pathogenesis of heart diseases and pathological responses which could provide novel sight into the pathogenesis of AF (Zhang et al., 2018). Unlike linear RNAs terminated with 5′caps and 3′tails, circRNAs are characterized by covalently closed loop structures which are presumably more stable and conserved, and may play important roles in many pathophysiological processes (Wang et al., 2016). Recently the role of circRNAs in cardiac disease conditions demonstrated their important functions as modulators of miRNA levels (Stepien et al., 2018). circRNAs may be a new kind of potential biomarkers and therapeutic targets, and their role in heart disease is becoming increasingly obvious.

To investigate the regulatory mechanism of circRNA in AF, especially the complex interactions among circRNA, microRNA and mRNA, we collected the heart tissues from three AF patients and three healthy controls and profiled their circRNA expressions with circRNA Microarray. The differentially expressed circRNAs were identified and the biological functions of their interaction microRNAs and mRNAs were analyzed. Our results provided novel insights of the circRNA roles in AF and proposed highly possible interaction mechanisms among circRNAs, microRNAs and mRNAs.

## Materials and Methods

### The circRNA Expression Profiles of Atrial Fibrillation Patients

We collected the heart tissues from three AF patients and three healthy controls. The clinical information of these six samples were given in Table 1. The circRNA expression profiles of these samples were measured with Arraystar Human circRNA Array V2 (8 × 15K, Arraystar). The arrays were scanned by the Agilent Scanner G2505C and analyzed with Agilent Feature Extraction software (version 11.0.1.1). The circRNAs presented in at least 3 out of 6 samples were retained. Finally, the expression levels of 12,515 circRNA probes were log2 transformed and quantile normalized. The circRNA expression profiles was given in Supplementary Table S1 and uploaded onto GEO (Gene Expression Omnibus) under accession number of GSE129409.

**Table 1:** Demographic characteristics of patients.

No.	Age (years)	Gender	NYHA	Coronary angiography	Complicated diseases	Duration of AF (years)	Operation
1	68	Male	II	Negative	Hypertension, cerebral infarction	3	Surgical AF ablation
2	75	Male	II	Negative	Hypertension, cerebral infarction	5	Surgical AF ablation
3	73	Male	II	Negative	Hypertension, cerebral infarction	4	Surgical AF ablation
4	35	Male	I	Negative	Negative	0	Healthy organ donors
5	35	Male	I	Negative	Negative	0	Healthy organ donors
6	40	Male	I	Negative	Negative	0	Healthy organ donors


*NYHA, New York heart association; INR, international normalized ratio; AF, atrial fibrillation.*

Written informed consent was obtained from patients before collection of the abandoned left atrial appendages. All experimental procedures were conducted in accordance with the Declaration of Helsinki and approved by the Ethics Committee of Shanghai East Hospital (approval no. 040-2017).

### Identify the Differentially Expressed circRNAs Between Atrial Fibrillation Patients and Healthy Controls

The statistical significance of differential expression between two groups was estimated with *t*-test using the R software limma package and further filtered with fold change. CircRNAs with *t*-test *p*-value smaller than 0.05 and fold change greater than 2 were considered as significant differentially expressed circRNAs.

### Construct the Integrative Regulatory Network of circRNAs, microRNAs, and mRNAs

The interactions between circRNAs and microRNAs play important roles for disease regulation (Ghosal et al., 2013). Some circRNAs contain microRNA sites and act as an endogenous microRNA “sponge” to adsorb and quench the normal biological functions of the microRNA (Lukiw, 2013). To discover such circRNA-microRNA interactions, we applied the TargetScan (Enright et al., 2003) and miRanda (Pasquinelli, 2012) to predict the microRNA targets within circRNAs. What’s more, we predicted the microRNA targets in mRNAs. At last, we constructed the genome wide integrative regulatory network of circRNAs, microRNAs and mRNAs.

### Analyze the Biological Functions of circRNAs, microRNAs, and mRNAs in Atrial Fibrillation

Since the functions of circRNAs are still poorly annotated, we investigated the functions of microRNAs interacted with differentially expressed circRNAs. These microRNAs may reflect the functions of differentially expressed circRNAs. We extracted 92 AF related microRNAs from HMDD (the Human microRNA Disease Database) v3.0 (Huang et al., 2018). These 92 AF related microRNAs were listed in Supplementary Table S2. If a circRNA interact with these microRNAs, it may be also related to AF. A complete interaction module of circRNAs, microRNAs and mRNAs with strong literature support from each angle will be a promising regulatory model for AF.

## Results

### The Differentially Expressed circRNAs Between Atrial Fibrillation Patients and Healthy Controls

If the *t*-test *p*-value was smaller than 0.05 and the fold change was greater than 2, a circRNA was considered as differentially expressed between AF patients and healthy controls. With these criteria, there were 537 up-regulated circRNAs and 199 down-regulated circRNAs in AF patients. These differentially expressed circRNAs between AF patients and healthy controls were listed in Supplementary Table S3. Since the sample size of the AF patients and healthy controls was too small, we did not use the FDR (False Discovery Rate) cutoff to identify the differentially expressed genes. But we still calculated the FDRs and the FDR was 0.556. We also calculated the mean and standard deviation (SD) of AF patients and healthy controls. For 537 up-regulated circRNAs, Mean_AF_ - SD_AF_ was always greater than Mean_Control_ + SD_Control_; for 199 down-regulated circRNAs, Mean_AF_ + SD_AF_ was always smaller than Mean_Control_ – SD_Control_. Such mean and SD results confirmed that there was difference between AF patients and healthy controls and the difference was greater than their variance.

We calculated the frequencies of microRNAs that targeted these up and down-regulated circRNAs, respectively. The top three most frequent microRNAs for up-regulated circRNAs were hsa-miR-597-3p that interacted with 18 up-regulated circRNAs, hsa-miR-136-5p that interacted with 16 up-regulated circRNAs and hsa-miR-103a-2-5p that interacted with 15 up-regulated circRNAs while the top three most frequent microRNAs for down-regulated circRNAs were hsa-miR-103a-2-5p that interacted with 11 down-regulated circRNAs, hsa-miR-4739 that interacted with 8 down-regulated circRNAs and hsa-miR-627-3p that interacted with 8 down-regulated circRNAs. These microRNAs may be the associated with the differentially expression pattern of circRNAs in AF.

### The circRNA–microRNA Interactions in Atrial Fibrillation

We extracted 92 AF related microRNAs from HMDD (the Human microRNA Disease Database) v3.0 (Huang et al., 2018). If the differentially expressed circRNAs we identified interact with these reported AF related microRNAs, they were more likely to be AF associated circRNAs. Therefore, we highlighted the differentially expressed circRNAs that interact with AF related microRNAs.

**FIGURE 1:**
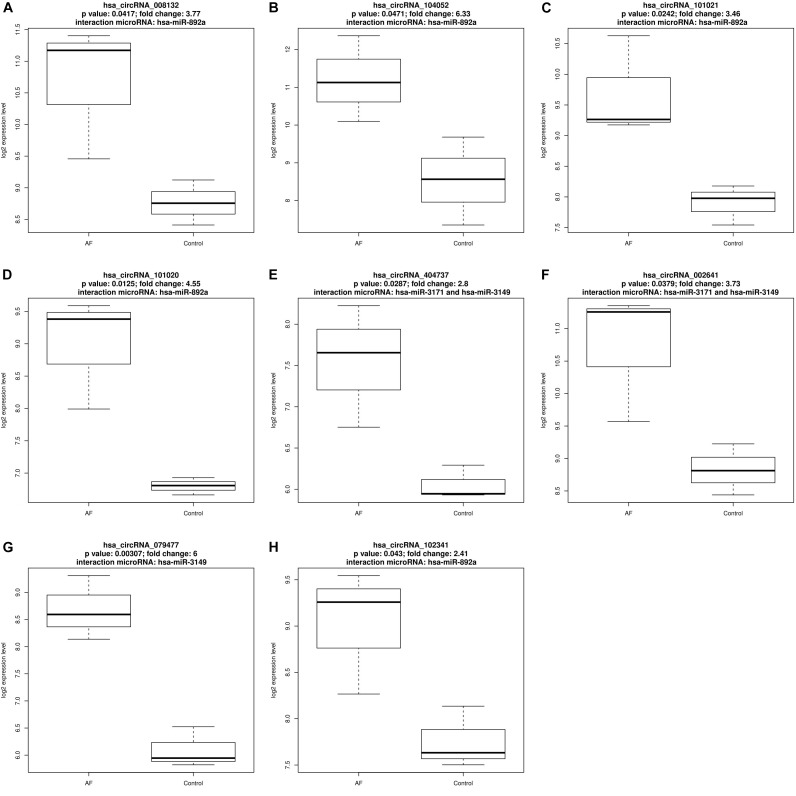
The expression pattern of the eight up-regulated circRNAs that interact with atrial fibrillation related microRNAs. **(A)** The expression pattern of up-regulated hsa_circRNA_008132 that interact with has-miR-892a; **(B)** The expression pattern of up-regulated hsa_circRNA_104052 that interact with has-miR-892a; **(C)** The expression pattern of up-regulated hsa_circRNA_101021 that interact with has-miR-892a; **(D)** The expression pattern of up-regulated hsa_circRNA_101020 that interact with has-miR-892a; **(E)** The expression pattern of up-regulated hsa_circRNA_404737 that interact with has-miR-3171 and has-miR-3149; **(F)** The expression pattern of up-regulated hsa_circRNA_002641 that interact with has-miR-3171 and has-miR-3149; **(G)** The expression pattern of up-regulated hsa_circRNA_079477 that interact with has-miR-3149; **(H)** The expression pattern of up-regulated hsa_circRNA_102341 that interact with has-miR-892a.

**FIGURE 2:**
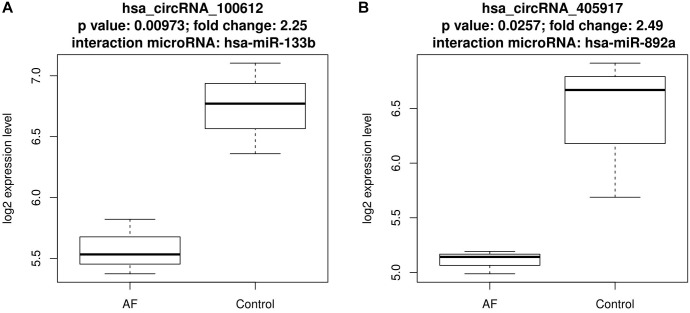
The expression pattern of the two down-regulated circRNAs that interact with atrial fibrillation related microRNAs. **(A)** The expression pattern of down-regulated hsa_circRNA_100612 that interact with has-miR-133b; **(B)** The expression pattern of down-regulated hsa_circRNA_405917 that interact with has-miR-892a.

There were eight up-regulated and two down-regulated circRNAs interact with AF related microRNAs. Figures 1, 2 plotted the expression pattern of these eight up-regulated circRNAs and these two down-regulated circRNAs, respectively.

Within the eight up-regulated circRNAs, five of them interacted with hsa-miR-892a, three of them interacted with hsa-miR-3149, two of them interacted with hsa-miR-3171. Within the two down-regulated circRNAs, one of them interacted with hsa-miR-892a while another interacted with hsa-miR-133b.

hsa-miR-892a interacted with both up-regulated and down-regulated circRNAs. A large number of differentially expressed circRNAs interact with hsa-miR-892a. Xu et al. (2016) reported that the expression level of has-miR-892a increased significantly from the early stage to the end stage of AF and it can be used as early diagnosis biomarker of AF.

has-miR-3149 had similar expression pattern with hsa-miR-892a and its expression level also increased in AF (Xu et al., 2016).

But hsa-miR-3171 had opposite expression pattern, its expression level decreased in AF (Xu et al., 2016).

The associations of hsa-miR-133b and AF has been reported by several studies but different expression patterns were observed. da Silva et al. found that hsa-miR-133b was up-regulated in acute new-onset AF patients with a 1.4-fold increased expression compared with well-controlled AF patients and control patients (da Silva et al., 2018). Li et al. (2012) reported that miR-133 was down-regulated in chronic AF canines. It was not clear whether such difference was cased by species or miR-133b functions differently at different stages of AF.

## Discussion

### The Potential Roles of has_circRNA_100612, has-miR-133b, and KCNIP1/JPH2/ADRB1 in Atrial Fibrillation

circRNA is a member of ncRNA family which could capture other RNA molecules and have recently shown as regulators of other proteins or RNAs including miRNAs. Recent studies have focused more attention on the potential of circRNAs to contribute toward disease etiology. And the expression pattern of circRNAs vary widely on different organism and cell types. Several recent studies have suggested that circRNAs may play essential roles in the initiation and development of cardiovascular diseases (Li et al., 2017). And miRNAs could regulate cardiac function through regulating the proliferation, migration, apoptosis, differentiation of cells during the progression of disease. A large number of literatures has reported association between miRNAs and AF related to remodel processes, and miRNAs might have important roles in signaling during the pathogenesis of AF (Flemming, 2014; Danielson et al., 2018). It has been shown that circRNAs may act as endogenous sponge RNAs to interact with miRNAs and influence the expression of miRNA target genes.

In our research, we found that circRNA_100612 which located on chromosome 10 could lead to AF by interacting with miR-133b. One of the target gene of miR-133b, KCNIP1, is a member of the family of cytosolic voltage-gated potassium (Kv) channel-interacting proteins and related to cardiac conduction pathway. In zebrafish, overexpression of KCNIP1 could lead to inducible AF. Genome-wide approach show a common 4,470 bp CNV in most AF patients indicated that KCNIP1 could be a genetic predictor of AF risk (Tsai et al., 2016).

Another important downstream target gene of miR-133b is JPH2 which have an important role in sarcoplasmic reticulum Ca^2+^ handling and modulation of ryanodine receptor Ca^2+^ channels. Knockdown JPH2 in mice was related to loss of junctional membrane complexes numbers, reduced Ca2+-induced Ca2+ release, and acute heart failure (van Oort et al., 2011). Mutation E169K in JPH2 could result in AF because of defective RyR2-mediated SR Ca2+ release events that representing a potential novel therapeutic target for AF (Beavers et al., 2013).

miR-133b could affect ADRB1 which is a member of the superfamily of cell surface receptors and has a great effect on the myocardium (Cresci, 2012; Pasquier et al., 2016). ADRB1 is also an effective target for pharmacotherapy in cardiovascular diseases, and β-blocking medications are acknowledged as first line agents for ventricular rate control in patients with AF (Chen et al., 2003; McMurray and van Veldhuisen, 2014).

### The Potential Roles of Differentially Expressed circRNAs, has-miR-892b, and GJA1 in Atrial Fibrillation

has-miR-892b interact with down-regulated has_circRNA_405917 and up-regulated hsa_circRNA_008132, hsa_circRNA_104052, hsa_circRNA_101021, hsa_circRNA_101020, hsa_circRNA_102341.

One important target gene of miR-892b is GJA1 which encodes the gap junction protein connexin 43 on chromosome 6q22.31 (Van Norstrand et al., 2012). A recent study using large-scale genotyping reported novel AF risk loci at or near GJA1. They found that SNPs associated with AF could influence the transcription of *GJA1* in both left atrial tissue and whole heart (Thibodeau et al., 2010; Sinner et al., 2014).

## Conclusion

**FIGURE 3:**
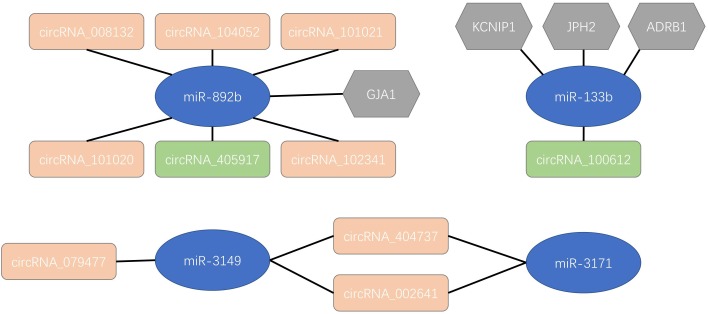
An integrative regulatory network model of circRNAs, microRNAs and target mRNAs in atrial fibrillation. The blue nodes were atrial fibrillation related microRNAs. The pink and green nodes were up and down-regulated circRNAs, respectively. The gray nodes were target mRNAs.

As the most common irregular heart rhythm disease, AF influence approximately 1–2% of the general population. The mechanisms behind AF pathogenesis are complex and remains elusive. circRNAs have been known as the key of developmental processes, regulation of cell function, pathogenesis of heart diseases and pathological responses which could provide novel sight into the pathogenesis of AF. By analyzing the circRNA expression profiles in AF patients and healthy controls, we identified 537 up-regulated circRNAs and 199 down-regulated circRNAs in AF patients. We investigated the interactions between these differentially expressed circRNAs and reported AF microRNAs. There were eight up-regulated and two down-regulated circRNAs interact with AF related microRNAs. By analyzing the functional interactions among circRNAs, microRNAs and target mRNAs, we proposed an integrative regulatory network model of circRNAs, microRNAs and target mRNAs for AF as shown in Figure 3. Our results provided novel insights of how circRNAs and microRNAs function in AF and the proposed regulatory network model of circRNAs, microRNAs and target mRNAs worth to be further studied and validated.

## Data Availability

The datasets generated for this study can be found in the GEO https://www.ncbi.nlm.nih.gov/geo/query/acc.cgi?acc=GSE129409.

## Ethics Statement

This study was carried out in accordance with the recommendations of (International Ethical Guidelines for Biomedical Research Involving Human Subjects), [Shanghai East Hospital Ethical (Tongji university school of medicine) committee]. The protocol was approved by the [Shanghai East Hospital Ethical (Tongji university school of medicine) committee]. All subjects gave written informed consent in accordance with the Declaration of Helsinki.

## Author Contributions

All authors listed have made a substantial, direct and intellectual contribution to the work, and approved it for publication.

## Conflict of Interest Statement

The authors declare that the research was conducted in the absence of any commercial or financial relationships that could be construed as a potential conflict of interest.

## Supplementary Material

TABLE S1 The circRNA expression profiles in three atrial fibrillation patients and three healthy controls.

TABLE S2 The 92 atrial fibrillation related microRNAs extracted from HMDD (the Human microRNA Disease Database) v3.0.

TABLE S3 These differentially expressed circRNAs between atrial fibrillation patients and healthy controls.
